# [^18^ F]FDG-PET imaging is an early non-invasive pharmacodynamic biomarker for a first-in-class dual MEK/Raf inhibitor, RO5126766 (CH5126766), in preclinical xenograft models

**DOI:** 10.1186/2191-219X-3-67

**Published:** 2013-09-16

**Authors:** Tetyana Tegnebratt, Li Lu, Lucy Lee, Valerie Meresse, Jean Tessier, Nobuya Ishii, Naoki Harada, Pavel Pisa, Sharon Stone-Elander

**Affiliations:** 1 Neuro Fogrp Stone-Elander, Neuroradiology, K8, MicroPET and Clinical Neurosciences, H3:00, Karolinska University Hospital, Karolinska Institutet, Stockholm SE-17176, Sweden; 2 Clinical Pharmacology, Hoffmann-La Roche Inc., Nutley 07110, NJ, USA; 3 Pharma Research & Early Development, Oncology, Hoffmann La Roche, Basel CH-4070, Switzerland; 4 Research Division, Chugai Pharmaceutical Co., Ltd, Kamakura 8144, Japan; 5 Pharma Research & Early Development, Oncology, Hoffmann-La Roche Inc., Schlieren, Switzerland

**Keywords:** Positron emission tomography, RO5126766, MEK inhibitor, Translational imaging

## Abstract

**Background:**

Positron emission tomography (PET) with [2-^18^ F]-2-fluoro-2-deoxy-D-glucose ([^18^ F]FDG-PET) was acquired at multiple time-points a) to monitor the early response to RO5126766 (CH5126766) in xenograft models b) to evaluate non-invasive small animal [^18^ F]FDG-PET imaging as a biomarker for MEK inhibitors for translation into dose-finding studies in cancer patients and c) to explore the underlying mechanism related to FDG uptake in tumors treated with RO5126766.

**Methods:**

[^18^ F]FDG uptake was studied in HCT116 (*K*-*ras*), COLO205 (*B*-*raf*) mutants and COLO320DM (wild type) xenografts from day 0 to 3 of RO5126766 treatment using a microPET Focus 120 and complemented with *in vitro* incubations, *ex*-*vivo* phosphor imaging and immunohistochemical (IHC) analyses.

**Results:**

In the HCT116 (*K*-*ras*) and COLO205 (*B*-*raf*) mutant xenografts, significant decreases in [^18^ F]FDG uptake were detected *in vivo* on day 1 with 0.3 mg/kg and *ex vivo* on day 3 with 0.1 mg/kg RO5126766. [^18^ F]FDG changes correlated with decreases in tumor cells proliferation (Ki-67) and with changes in expression levels of GLUT1. No effects were observed in drug resistant COLO320DM cells. The cellular fractionation and Western blotting analyses suggested that the change of [^18^ F]FDG uptake associated with RO5126766 is due to translocation of GLUT1 from membrane to cytosol, similar to the results reported in the literature with EGFR tyrosine kinase inhibitors, which also target the MAPK pathway.

**Conclusions:**

RO5126766 inhibition resulted in a rapid time - and dose - dependent decline in [^18^ F]FDG uptake in both mutant xenografts. These results strongly resemble the clinical observations obtained with MEK/Raf inhibitors support the use of preclinical [^18^ F]FDG-PET as a translational tool for decision support in preclinical and early clinical development of MEK inhibitors.

## Background

The Ras/Raf/mitogen-activated protein kinase kinase (MEK)/extracellular signal-regulated (ERK) cascade transmits signals from the cell surface receptors to the nucleus and regulates cell cycle progression, cell proliferation, survival, differentiation and transformation. The genetic mutations in many of the components in this pathway have been found to be associated with cancers. The Ras/Raf/MEK/ERK pathway has a well-defined role in cancer biology and has become an important target in the development of cancer therapeutics [1-3]. Many drugs targeting the ligand-activated receptor tyrosine kinases and their downstream effectors such as Ras, Raf and MEK are currently being tested in clinical trials [4-7].

A major drawback in the clinical testing of the new drugs is the lack of pharmacodynamic biomarkers at early stage clinical trials. Non-invasive imaging techniques have demonstrated a potential for accelerating the drug development process by assessing therapeutic response and early identification of responders [8-10]. Positron emission tomography (PET) imaging with the fluorine-18 labeled glucose analog 2-fluoro-2-deoxy-D-glucose ([^18^ F]FDG-PET) is increasingly being included as a new functional endpoint in phase I to III clinical trials in oncology, in addition to conventional endpoints such as toxicity and decreases in tumor size. Many of these clinical studies have reported that [^18^ F]FDG-PET imaging can be successfully used for monitoring the efficacy of a range of targeted therapies [11-13].

MEK has a critical position in the Ras/Raf/MEK/ERK pathway with few direct upstream activators (e.g. Raf) and few downstream targets (e.g., ERK). The successful development of MEK inhibitors and their evaluations in various clinical trials is well summarized in recent reviews [14,15]. [^18^ F]FDG-PET imaging has been included as a therapeutic read-out in several of these studies [16-18]. PET/CT imaging was also used as a primary therapeutic endpoint for sorafenib, inhibitor of Raf kinase activity [19].

Despite these applications of [^18^ F]FDG-PET as an efficacy biomarker for MEK inhibitors in humans, very few preclinical studies have been reported. Early studies demonstrated instead the utility of the thymidine analog 3’-deoxy-3’-^18^ F-fluorothymidine (FLT) for therapeutic monitoring of the MEK inhibitor PD0325901 in a ^*V600E*^*B*-*raf* mutant SK-MEL-28 melanoma model [8,20]. [^18^ F]FDG-PET was used to evaluate inhibitors of PI3K/AKT/mTOR and epidermal growth factor receptor (EGFR) pathways either alone or in combination with a MEK inhibitor. For instance, PET/CT together with magnetic resonance imaging demonstrated the synergistic effects of NVP-BEZ235, a dual PI3K/mTOR inhibitor, and ARRY-142886 (AZD6244/selumetinib), an allosteric MEK inhibitor, on *K*-*ras* mutated tumor in a genetically engineered mouse model of lung adenocarcinoma [21].

The value of using [^18^ F]FDG-PET as an early surrogate marker has been demonstrated in several preclinical models. Studies in xenografts sensitive to gefitinib, an EGFR tyrosine kinase inhibitor (EGFR-TKI), revealed up to a 55% decrease in [^18^ F]FDG uptake within 48 hours after start of treatment [9]. [^18^ F]FDG-PET could also be a surrogate marker for the efficacy of erlotinib, another EGFR-TKI, in preclinical human head and neck carcinoma models [22] and of the c-KIT inhibitor, imatinib, in models with activating *c*-*KIT* mutations in gastrointestinal stromal tumors (GISTs). Preclinical PET imaging revealed that [^18^ F]FDG uptake in tumors sensitive to the drug was significantly reduced as early as 4 hours after imatinib treatment while no response was observed in resistant tumors [23].

The main goal of our study was to explore whether the effects of MEK/Raf inhibitors in humans revealed with [^18^ F]FDG-PET could be replicated in animals and whether [^18^ F]FDG-PET can therefore be used in preclinical models as an endpoint for early detection of therapeutic activity and dose-finding studies for this class of inhibitors. For this purpose, we have used RO5126766, a first-in-class orally active and highly selective dual protein kinase inhibitor, specific for Raf and MEK. RO5126766 is a novel chemical class allosteric inhibitor of MEK activity and prevents MEK from phosphorylation by Raf through stable Raf-MEK complex formation. RO5126766 inhibits ERK signalling more effectively that a standard MEK inhibitors. It suggests a new therapeutic approach for *ras* tumors by blocking feedback activation of ERK signalling [24]. RO5126766 has shown potent *in vivo* anti-tumor efficacy in diverse human tumor xenografts models and has recently been evaluated in a phase I dose-escalation study in humans in which [^18^ F]FDG-PET was included as one of the biomarker assessments [18,25]. Our results show that in vivo [^18^ F]FDG-PET imaging of preclinical tumor models can be used to successfully monitor therapeutic response to MEK inhibition.

## Methods

### Cell culture and reagents

The human colon cancer cell lines HCT116, COLO205 and COLO320DM were purchased from the American Type Culture Collection (ATCC). All cells were maintained in the designated media and indicated concentrations of heat-inactivated fetal bovine serum (Gibco) and L-glutamine (Sigma) according to the ATCC recommendations. Cells were grown at 37°C in an atmosphere of 5% CO_2_. RO5126766 (CH5126766) was synthesized in Chugai Pharmaceuticals Co., Ltd. For *in vitro* and *in vivo* studies, the drug was dissolved in DMSO (Wako Chemicals GmbH) to yield a 2.5 mg/mL stock solution concentration and stored at -20°C. The solutions of RO5126766 used for *in vitro* and *in vivo* experiments were freshly prepared on each experimental day. The vehicle and RO5126766 stock solutions were diluted 1:20 with the diluent (10.5% aqueous solution of 2-hydroxypropyl-β-cyclodextrin (Celdex HP- β-CD, HPCD, Sigma)) on each dosing day.

### [^18^ F]FDG uptake *in vitro*

[^18^ F]FDG uptake was determined in untreated HCT116, COLO205 and COLO320DM cells as well as treated with vehicle only as a control or RO5126766 at indicated concentrations. 1×10^5^ cells/well were seeded in 6-well plates (Costar®) together with appropriate doses of RO5126766 for indicated times. Cell culture medium was changed to glucose-free and 0.37 MBq of [^18^ F]FDG was added to each well and incubated for 1 hour in 5% CO_2_ atmosphere at 37°C. The cells were washed three times with ice cold PBS and radioactivity was measured using a 1480 Automatic gamma counter Wizard3 (Perkin Elmer). [^18^ F]FDG incorporation was determined and expressed relative to total protein concentration. Protein content was determined using the Thermo Scientific Pierce BCA Protein Assay Kit (Waltham, USA).

### Cellular fractionation and Western blotting

Plasma membrane fractionation was performed using a membrane protein extraction kit from BioVision. According to the manufacturer's recommendations, 5×10^8^ HCT116 cells were used for protein extraction. The purity of the plasma membrane protein fraction was assessed by Western blot analysis of the plasma membrane marker (mouse monoclonal antibodies to Na^+^, K^+^-ATPase from Abcam). For Western blot analysis, 15 μg of plasma membrane-associated or of the cytosol proteins were separated on 4-12% poly-acrylamide gels (Invitrogen) and Western blotting was performed. The rabbit polyclonal antibodies to glucose transporters GLUT1 and GLUT3 were from Abram (Cambridge, UK). (Secondary HRP-linked anti-mouse and anti-rabbit IgG were from Cell Signaling (In Vitro Sweden AB, Stockholm, Sweden). Bands were visualized with Western blotting Luminol Reagent (Santa Cruz). The images were captured using a LAS-1000 from Fujifilm or exposed to X-ray film (Fujifilm, Tokyo).

### Xenograft tumor models

Female athymic nude (nu/nu) mice, age 5–6 weeks (18-22 g) were purchased from Scanbur AB (Sollentuna, Sweden). Animal care, handling and health monitoring were carried out in accordance with the Guidelines for Accommodation and Care of Laboratory Animals. All animal experiments were performed in accordance with protocols approved by the Institutional Animal Care committee.

For the tumor xenografts, 5×10^6^/mouse human cancer colon carcinoma cells were inoculated subcutaneously in the right flank of Balb-nu/nu mice. Once tumors were established (150–200 mm^3^), mice were randomized into groups with similar mean tumor volumes at the start of the study. Tumor volume and body weight were measured two times per week. Tumor volumes were determined with digital caliper using the formula (tumor length × width^*2*^)/2. Tumor growth inhibition (TGI) was calculated using the following formula: TGI = [1 − (*T* − *T*_*0*_)/(*C* − *C*_*0*_)] × 100, where *T* and *T*_*0*_ are the mean tumor volumes on a specific experimental day and on the first day of treatment, respectively, for the experimental groups and likewise, where *C* and *C*_*0*_ are the mean tumor volumes for the control group. The daily administration of RO5126766 was performed orally at doses 0.1, 0.3 and 1.0 mg/kg. The doses were selected based on the results of preliminary studies. The maximal tolerated dose (MTD) was defined as the maximum dose associated with <20% weight loss and no toxic deaths. The MTD in the three xenograft models was 1.5 mg/kg for RO5126766.

### MicroPET imaging

MicroPET imaging was performed by standard protocols as described previously [26]. Mice were fasted for 6–8 hours prior to start of imaging session [27]. [^18^ F]FDG (obtained as an aliquot from daily clinical productions at Karolinska University Hospital, 7–8 MBq per mouse, maximum volume of 200 μl) was administered to awake, warmed (37°C) mice by a bolus injection via the tail vein. Forty to sixty minutes after the tracer injection, the mice were anaesthetized with isoflurane, controlled by an E-Z anaesthesia vaporizer (5% initially and then 1.5% to maintain anaesthesia, blended with 7:3 air/O2 and delivered through a Microflex non-rebreather mask from Euthanex Corporation, Palmer, PA). The mice were placed on a heating pad (37°C) on the camera bed, with most of the body in the field-of-view (7.68 cm). Emission data were collected for 20 minutes in list mode with MicroPET Focus 120 scanner (CTI Concorde Microsystems). Data were processed using MicroPET Manager (CTI Concorde Microsystems). PET data were acquired in fully three-dimensional (3-D) mode and images were reconstructed by standard 2-D filtered back projection using a ramp filter. The matrix size of the reconstructed images was 128 × 128 × 95 with a spatial resolution of 1.3 mm. Data were corrected for randoms, dead time and decay. Standard Uptake Values (SUV) were calculated for 3D regions of interest (ROI), using Inveon Research Workplace software (Siemens Medical Solutions). Tumor ROIs drawn on the images employed a 75% threshold of the maximum intensity voxel. The ROI counts were normalized to the injected dose and body weight and converted to SUV. The drug effect on tumor metabolism was estimated as%SUV_max_ change day 1, 2, or 3 compared to day 0 (baseline).

### *Ex*-*vivo* phosphor imaging

Immediately after the last MicroPET scan, the animals were sacrificed and their tumors were rapidly frozen. Tumor slices (20 μm thickness) were obtained using a cryomicrotome (CM 3050S, Leica Microsystems, Wetzlar, Germany) and were placed on Superfrost Plus microscope slides (Menzel-Glaser, Germany). The sections were placed in a BAS exposure cassette with a 2325 imaging plate (Fujifilm Corporation, Japan) and exposed for at least 1 hour. The quantitative autoradiography data (photo-stimulated luminescence, PSL, unit/mm^2^) were normalized to the injected dose and body weight.

### Immunohistochemistry

Tumor slices were obtained as described above. Frozen tumor slices sections were kept at −80°C until needed. For IHC, frozen sections (20 μm) were air dried and fixed for 10 minutes with ice-cold acetone. The slides were incubated in 2.5% normal horse blocking serum in PBS before primary antibodies, rabbit polyclonal to Ki-67 (Abcam), were added followed by incubation for 1 hour. As the secondary antibody, ImmPRESS™ reagent, anti-rabbit Ig, peroxidase (Vector) was used. The staining’s were visualized by using an ImmPact™ DAB substrate kit (Vector). The counter stain was performed in methyl green for 2 minutes at room temperature. The images were analyzed with an Olympus UC30 digital color camera (*Olympus Color* Management Technology) and Cell Imaging software for Life Sciences microscopy.

### Statistical analysis

Statistical significance was examined by Student *t* test. *P*-values less than 0.05 were considered a statistically significant difference.

## Results

### Effects of RO5126766 on cellular determinants of the uptake and retention of [^18^ F]FDG

**Figure 1:**
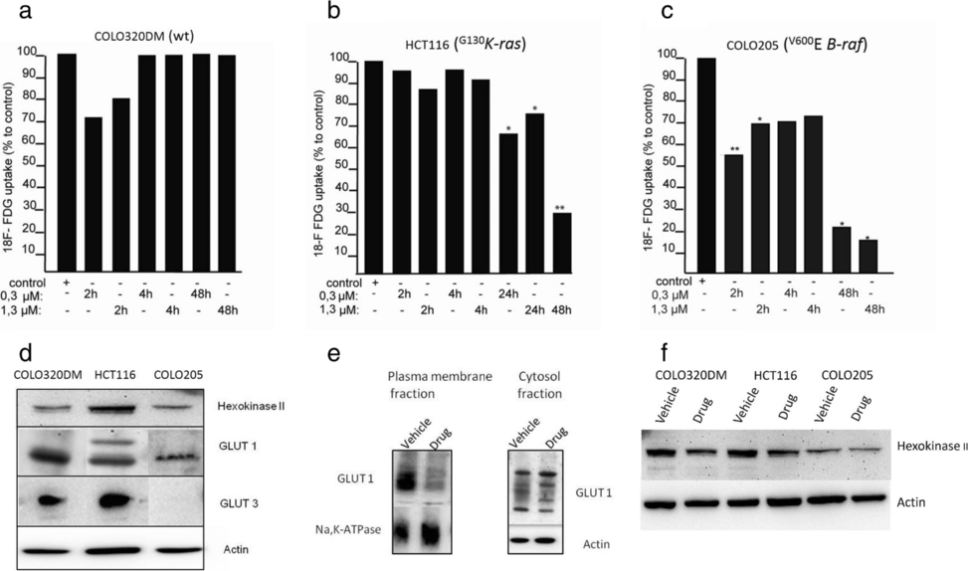
**Effects of RO5126766 on cellular determinants of the uptake and retention of [**^**18**^ F]FDG: (a) COLO320DM (wild type); (b) HCT116 (*K-ras* mutant) (c) COLO205 (*B-raf*) mutant) cells were treated with RO5126766 for the indicated time- and dosage range and FDG uptake was assessed *p<0.05; **p<0.01; (d) Western blotting analysis of the GLUTs and hexokinase II levels in three cell lines, actin is a loading control; (e) GLUT1 expression in plasma-membrane and cytosol fractions of HCT116 (*K*-*ras*) cells, treated with RO5126766 (1.3 μM). Na^**+**^**, K**^**+**^**-ATP**_**ase **_**and actin protein levels in the cell membrane and cytosolic fractions (respectively) served as the fractions purity and loading controls; (f) Western blotting analysis of the hexokinase II activity in three cell lines, treated for 24 hours with RO5126766, 1.3 μM.** Actin is a loading control.

We first investigated *in vitro* the feasibility of using two RO5126766 sensitive cell lines, HCT116 (^*G13D*^*K*-*ras*) and COLO205 (^*V600E*^*B*-*raf*), and one resistant cell line COLO320DM (no *K*-*ras* and *B*-*raf* mutation) for [^18^ F]FDG-PET imaging. The study revealed variations in glucose utilization between the three cell lines. The highest cellular glucose uptake was observed in COLO320DM cells and lowest in COLO205 (Additional file 1: figure S1). The RO5126766 at the concentration of 0.3 μM was a minimal dose demonstrated reduction of ERK/MEK phosphorylation to undetectable levels in HCT116 (*K*-*ras*) cells after 2 hours of the treatment start [24]. Variations in [^18^ F]FDG uptake in tumor cells exposed to RO5126766 were subsequently examined with 0, 0.3 or 1.3 μM of RO5126766 up to 48 hours. There was no significant change in the cellular accumulation of [^18^ F]FDG in the drug-resistant COLO320DM during treatment (Figure 1a). In HCT116 cells, a significant reduction in [^18^ F]FDG uptake was observed after 24 h of treatment (67.1%, *p* < 0.05, Figure 1b) and not at earlier time points. In contrast, in drug-sensitive COLO205 cells, a dose-dependent decrease in [^18^ F]FDG uptake was observed as early as after 2 hours of treatment compared to control (54.8%, *p* < 0.01, Figure 1c).

In order to identify the cellular components determining the uptake and retention of [^18^ F]FDG in these cell lines, the expression levels of glucose transporters (GLUTs) and hexokinases were analyzed by Western blotting. GLUT1 was detected in all cell lines. GLUT3 was expressed in COLO320DM and HCT116 but not in COLO205 cells. Hexokinase II was expressed in all cell lines (Figure 1d). To further examine possible mechanisms behind the RO5126766-induced changes in [^18^ F]FDG uptake, we used the HCT116 cell line because of its higher basal glucose utilization. We detected significant decreases in the expression of the cellular transmembrane protein GLUT1 in the plasma membrane fraction after 24 hours of treatment with 1.3 μM of RO5126766, compared to the vehicle treated cells. In parallel, an increase of GLUT1 in the cytosol fraction was observed during treatment (Figure 1e). We did not detect significant changes in hexokinase II activity during the treatment of HCT116 cells with 1.3 μM of RO5126766 for 24 hours (Figure 1f).

### Anti-tumor activities of RO5126766 and FDG-PET imaging results in human colon carcinoma xenografts in balb nu/nu mice

**Figure 2:**
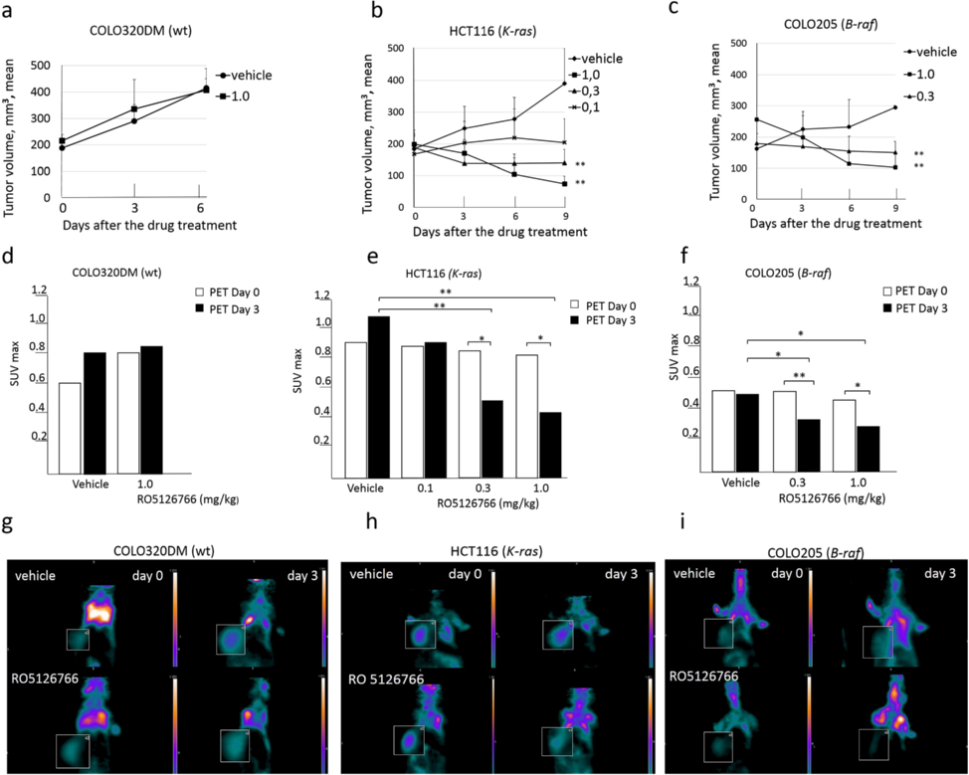
**Anti-tumor activities of RO5126766 and FDG-PET imaging results in human colon carcinoma xenografts in Balb nu/nu mice.** Mice bearing **(a)** COLO320DM (wt) **(b)** HCT116 (*K*-*ras*) and **(c)** COLO205 (*B*-*raf*) mutant tumor xenografts were orally administered once daily for 9 days with vehicle, 0.1, 0.3 or 1.0 mg/kg of RO5126766. The tumor sizes are mean ± SD (n = 10). Error bars = standard error, **p < 0.01. FDG-PET imaging was performed at baseline (day 0) and day 3 after the treatment with RO5126766 in **(d)** COLO320DM (wt) (only high dose) **(e)** HCT116 (*K*-*ras*) and **(f)** COLO205 (*B*-*raf*). The SUV_max_ values are mean ± SE (n = 6). *p < 0.05, **p < 0.01. Representative PET images (coronal sections) of **(g)** COLO320DM (wt) **(h)** HCT116 (*K*-*ras*) and **(i)** COLO205 (*B*-*raf*) tumors on days 0 (baseline) and 3 of the treatment with RO5126766, 1 mg/kg. Tumors are shown in the boxed areas, twofold magnifications. All images scaled to the same color scale.

*In vitro* experiments demonstrated that RO5126766 treatment resulted in dose-dependent decreases in [^18^ F]FDG uptake for both *K*-*ras* and *B*-*raf* mutants, but not for COLO320DM, the resistant cell line. Furthermore, PET imaging of antitumor activities of RO5126766 and quantification of early response in the three colorectal cancer xenograft models were evaluated on days 0 and 3 of the treatment. Once the tumors were established (approximately 0.2 cm^3^) and mice were divided into treatment groups (n = 10/group) and treatment was initiated with vehicle and RO5126766 at 0.1, 0.3 or 1.0 mg/kg daily oral gavage for 9 days. RO5126766 treatment (1.0 mg/kg) did not inhibit growth of the COLO320DM tumors (Figure 2a) and these mice were therefore sacrificed after 6 days of treatment when the tumors had reached the size limits allowed by research ethics. In contrast, RO5126766 treatment showed dose-dependent tumor growth inhibition (TGI) in the mice with xenografts of both the mutant models. In HCT116 (*K*-*ras*) tumor xenografts the treatment resulted in 80% TGI (0.1 mg/kg), 119% TGI (0.3 mg/kg, *p* < 0.01) and 157% TGI (1.0 mg/kg, *p* < 0.01) (Figure 2b). In the COLO205 (*B*-*raf*) mutant tumor xenografts TGI’s of 120% (0.3 mg/kg, *p* < 0.01) and 190% (1.0 mg/kg, *p* < 0.01) (Figure 2c) were achieved.

[^18^ F]FDG uptake was measured in tumors of mice treated with RO5126766 at 0.1, 0.3 or 1.0 mg/kg versus vehicle from day 0 (baseline) to day 3. PET imaging revealed no significant effect on [^18^ F]FDG uptake in COLO320DM tumors during the treatment (Figure 2d). In contrast, RO5126766 treatment HCT116 (*K*-*ras*) tumors demonstrated significant decrease in metabolic activity on day 3, compared to day 0 (*p* < 0.05 at 0.3- and 1.0 mg/kg doses and vehicle (*p* < 0.01). No significant effect was observed in the 0.1 mg/kg dosing group (Figure 2e). Despite the low basal [^18^ F]FDG-uptake in COLO205 tumor xenografts, we could detect inhibition by RO5126766 at both 0.3 mg/kg (*p* < 0.05) and 1.0 mg/kg (*p* < 0.05) doses. Representative coronal PET images in Figure 2g-i demonstrate the FDG uptake observed in COLO320DM (wt) tumors, HCT116 (*K*-*ras*) and COLO205 (*B*-*raf*), respectively, at days 0 and 3 of treatment with vehicle versus RO5126766 (only 1.0 mg/kg dose shown).

**Figure 3:**
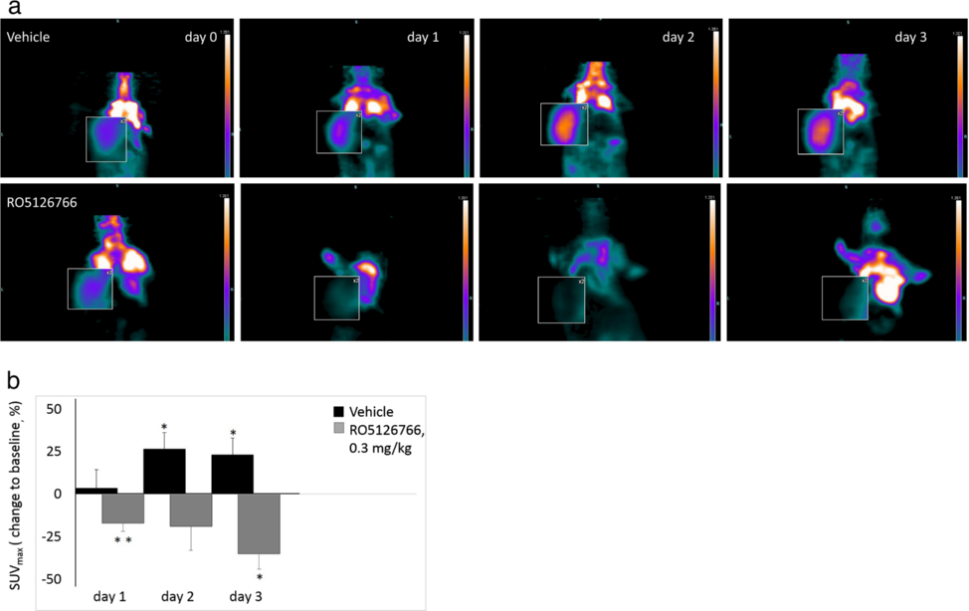
**Serial FDG-PET imaging results in HCT116 (*K*-*ras*) tumors at early times after treatment with RO5126766, 0.3 mg/kg. (a)** Coronal PET images from one mouse presenting vehicle - (upper panel) and RO5126766 treated group on days 1, 2 and 3 of treatment. Tumors are shown in the boxed area, twofold magnification. **(b)** Comparison of changes in [^18^ F]FDG uptake from baseline to day 1, day 2 and day 3 of treatment, presented as% change SUV_max_ value to baseline (*p < 0.05; **p < 0.01). The SUV_max_ values are mean + SE (n = 5).

The HCT116 tumors demonstrated higher basal [^18^ F]FDG-uptake and were usually more readily distinguished from background tissues than COLO205 tumors. Therefore, additional PET scans and *ex*-*vivo* phosphor imaging for earlier time points than day 3 and one lower dose were performed using HCT116 xenografts. Serial microPET imaging revealed significant decreases in tumor [^18^ F]FDG uptake as early as on day 1 after administration of 0.3 mg/kg RO5126766 (Figure 3a, representative coronal images). Maximum reductions of [^18^ F]FDG-uptake tumors treated with RO5126766, 0.3 mg/kg on day1, day2 and day3 were 17% (*p* < 0.01), 19% (*p* = 0.15) and 35% (*p* < 0.05) compared with baselines values, respectively (Figure 3b).

### Phosphor imaging of [^18^ F]FDG uptake and immunohistochemistry in excised tissue sections

**Figure 4:**
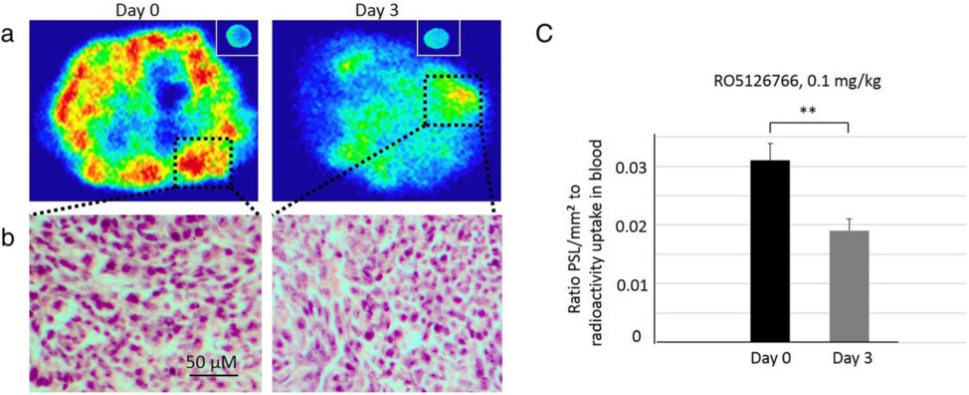
**Ex-vivo phosphor imaging and quantitative autoradiography data.** Ex-vivo phosphor imaging of radioactivity distribution **(a)** on day 0 and day 3 of the treatment HCT116 (*K*-*ras*) tumor-bearing mice with RO5126766, 0.1 mg/kg. The small image in the right corner is the radioactivity in a blood sample (20 μL) from each individual mouse used as a reference to normalize the tumor radioactivity uptake. In box: vital tumor area with highest radioactivity uptake and **(b)** corresponding H&E staining. **(c)** Quantitative autoradiography data presented as ratio of photo-stimulated luminescence, PSL, unit/mm^2^ to radioactivity uptake in the blood sample and normalized to the injected dose and body weight (**p < 0.01, n = 6).

In the mice bearing HCT116 xenografts that received the lowest dose RO5126766 (0.1 mg/kg), statistically significant changes in [^18^ F]FDG uptake could not be detected by PET on day 3 (Figure 2e). As a complement to PET, *ex*-*vivo* phosphor imaging at higher resolution and sensitivity was also performed (Figure 4a,b, representative images). Tumors were sectioned after PET imaging and exposed to phosphor imaging plates together with a blood sample from the same mouse. We observed higher radioactivity concentrations ([^18^ F]FDG uptake) in tumors on day 0 compared to day 3 of treatment (Figure 4a). Hematoxylin & eosin examination revealed no difference in examined tumors morphology (Figure 4b). The result was confirmed by statistical analysis of the [^18^ F]FDG uptake in a larger population of mice (n = 6, *p* < 0.01) (Figure 4c). However, the *ex vivo* technique of course only gave one time point per animal and could not be used for monitoring individual responses over time.

### Histopathology in RO5126766 treated mice

**Figure 5:**
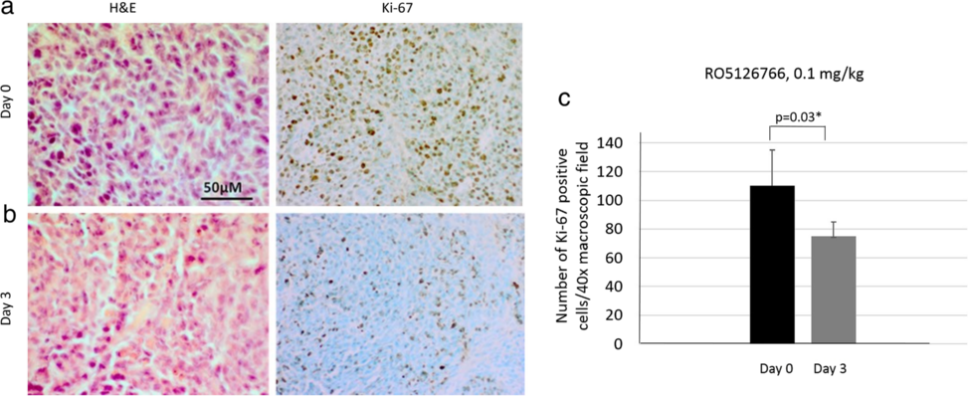
**Representative immunohistochemical images of Ki-67 positive cells in HCT116 (*K*-*ras*) xenografts in mice treated with a lowest dose RO5126766 (0.1 mg/kg). (a)** tumor morphology, visualized with H&E staining (left image) and corresponding Ki-67 positive staining (brown cells, right image) before the treatment start (baseline) and **(b)** after 3 days of treatment with RO5126766 (0.1 mg/kg). **(c)** Quantitative assessment of proliferating index of tumor cells in relation to RO5126766 treatment based on IHC staining of Ki-67 antigen of excised, acetone fixed frozen tumor tissue (*p < 0.05, n = 3).

The tumors that were subjected to *ex*-*vivo* phosphor imaging and excised from mice treated with 0.1 mg/kg RO5126766 on day 3 were also analyzed histologically (hematoxylin & eosin, H&E) and immunohistochemically (Ki-67) (Figure 5a, b). The difference in [^18^ F]FDG uptake in tumors correlated with their proliferative activity as detected with Ki-67 antigen. Figure 5c shows the number of proliferating cells on day 0 (baseline) and day 3 after therapy.

## Discussion

In this study we have demonstrated the feasibility of using [^18^ F]FDG-PET imaging as an early surrogate endpoint for monitoring biological and anti-tumor activity of MEK/Raf inhibitors given for the treatment of human cancers. We first used *in vitro* experiments to investigate whether RO5126766 effects on sensitive tumor cells would be accompanied by changes in the uptake of labeled glucose. We found that [^18^ F]FDG uptake was reduced in a dose (0.3-1.3 μM) - and time (0-48 h) - dependent manner in HCT116 and COLO205 tumor cells carrying *K*-*ras* and B-*raf* mutations, respectively, whereas RO5126766 did not affect [^18^ F]FDG uptake in COLO320DM cells, which has no mutation in these two genes and no apparent levels of phospho-MEK and phospho-ERK in the cells. Both mutant tumor cell lines demonstrated metabolic sensitivity to the drug, confirming their feasibility for [^18^ F]FDG-PET imaging of RO5126766 efficacy. However, *in vitro* results showed variations in basal [^18^ F]FDG-uptake among three cell lines, with the lowest levels observed in COLO205.

The transport of glucose through the cell membrane via glucose transporter proteins and its subsequent intracellular phosphorylation by hexokinases are key steps required for its cellular accumulation [28]. The expression levels of glucose transporters and hexokinases are changed in many cancers [29,30]. Chung *et al*. [31] suggested that increased numbers of glucose transporters at the plasma membrane of cancer cells may be a cause of increased [^18^ F]FDG uptake, at least in colon cancers. Yun *et al*. [32] reported that GLUT1 expression levels were consistently upregulated and that glucose uptake was enhanced in *K*-*ras* and *B*-*raf* mutated cells compared to wild type cells. Drug-induced changes in [^18^ F]FDG uptake together with the expression levels of GLUTs and hexokinases in tumor cells may therefore serve as good predictors for how well [^18^ F]FDG-PET can be used for monitoring response *in vivo* in xenografts from a particular cell line. We observed that GLUT1 expression levels decreased in the plasma membrane and increased in the cytosol fractions of HCT116 cells treated with RO5126766. These results are indicative of a RO5126766-induced translocation of GLUT1 from the plasma membrane to the cytosol, which could be a possible mechanism behind the observed reductions in [^18^ F]FDG uptake in the drug-treated cells. Similar translocation effects on glucose transporters have been reported for the EGFR inhibitors, gefitinib [9] and erlotinib [22].

This study shows that in RO5126766-sensitive cells MEK and Raf inhibition results in a rapid decrease in [^18^ F]FDG uptake. In contrast, in COLO320DM resistant cells (no detectable pMEK and pERK levels, suggesting no activation of RAF and MEK), RO5126766 did not affect the glucose uptake. These results support the applicability of FDG-PET as a pharmacodynamic biomarker for MEK/Raf inhibitors.

*In vivo* imaging revealed significant reductions in [^18^ F]FDG uptake as early as after 1 day of treatment with 0.3 mg/kg of RO5126766 in both HCT116 and COLO205 xenografts (37% and 43% , respectively). The FDG change paralleled but preceded the drug-induced reductions in xenograft sizes. In HCT116 tumors the [^18^ F]FDG uptake was increasingly reduced over time (for example, at the dose of 0.3 mg/kg, for 17% at day 1, 19% at day 2 and 35% on day 3) and exposure-dependent, showing a decrease from baseline on day 3 (range from 97 to 52% at the doses range 0.1-1.0 mg/kg) compared to an increase in vehicle treated group (126%). These observations are consistent with reports elsewhere of early decreases in [^18^ F]FDG uptake for mTOR inhibition in experimental lymphoma model [33], and for combined PI3K/mTOR (PF-04691502) and MEK (PD-0325901) inhibitors in a K-*ras*^*G12D*^; *Pten* mutated mouse model of ovarian cancer [34].

Partial volume effects associated with imaging tissues close in size to the 1–2 mm resolutions of small animal PET and the heterogeneous nature of tumors including varying amounts of necrosis and non-tumor tissue can affect *in vivo* quantifications [35]. In order to account for these limitations and further validate the results obtained, we used PET imaging combined with *ex vivo* phosphor imaging to evaluate minimal effective doses and times for RO5126766 efficacy in the tumor xenografts. With an order of magnitude higher resolution, *ex vivo* phosphor imaging can serve as a useful single time point complement to the longitudinal *in vivo* information obtained from small animal PET imaging [36]. Using *ex vivo* phosphor imaging, reductions in [^18^ F]FDG uptake could also be detected even for the lowest administered dose (0.1 mg/kg) on day 3 of treatment in HCT116 tumors. We also observed high [^18^ F]FDG uptake in necrotic-free tumor fractions of vehicle treated mice, compared to low uptake in tumors of the drug treated animals. Thus *ex vivo* tissue sampling was helpful in defining the dose- and time- dependency while using microPET to examine MEK inhibition at selected doses over time.

[^18^ F]FDG uptake reductions in drug-treated tumors correlated with decreased number of proliferating cells in RO5126766 treated tumors measured with Ki67. This is in agreement with recently published studies suggesting that cellular proliferation and metabolism are tightly linked processes that share common regulatory pathways in tumour cells [37]. It has been shown [38,39] that some oncoproteins (such as Ras, c-Myc, Akt) participate in the control of cancer cell metabolism. Therefore, in addition to the metabolic studies with [^18^ F]FDG, we also investigated the proliferation status of the tumor cell lines during treatment with RO5126766. In this study, we observed significant decreases in the number of proliferating cells in HCT116 tumors upon exposure to the drug, which supports also the use of proliferation PET tracers such as [^18^ F]FLT [40] for evaluating the anti-proliferative activity of RO5126766.

Both EGFR-TKIs, for which [^18^ F]FDG-PET as already demonstrated utility in monitoring efficacy [8], and MEK/Raf inhibitors are targeting the MAPK pathway. EGFR inhibitors block the initiation of the pathway at the upstream receptor site while MEK/Raf inhibitors block pathway signaling at one of the effector sites downstream from the receptor. In our study, we observed similarities between the effect obtained with the MEK/Raf inhibitor and the one reported in the literature with EGFR inhibitor. In both cases drug treatment was associated with a reduction of FDG-PET uptake and in both cases this was accompanied by a translocation of GLUT1 from the plasma membrane to the cytosol. The similarity observed between MEK/Raf and EGFR inhibitors provides further evidence that cellular glycolytic metabolism as measured by the uptake and retention of [^18^ F]FDG provides an effective downstream pharmacodynamic read-out for therapeutic strategies targeting inhibition of signaling components of the MAPK pathway.

These preclinical studies were performed in parallel with phase 1 dose escalation clinical studies of the dual inhibitor, RO5126766, in patients with locally advanced and/or metastatic solid tumors without specific genotype. The reduction in FDG uptake observed in the current pre-clinical study mimics the results observed clinically. In both studies, the decrease in FDG uptake was dose dependent with similar overall reduction in FDG uptake (approximately 35% on day 3 in drug-sensitive xenograft models (HCT116, COLO205) compared to 28% on day 15 in patients with melanoma [18]).

## Conclusions

Our preclinical PET imaging studies support the use of [^18^ F]FDG-PET imaging as an early pharmacodynamic biomarker in preclinical studies of MEK and Raf inhibitors, with strong decreases in SUV_max_ observed as early as 24 hours post treatment. The decrease in [^18^ F]FDG uptake was dose-dependent and increased with treatment exposure, therefore strongly paralleling and supporting the observations obtained with this class of compounds in patients [18,25]. The effect in [^18^ F]FDG uptake *in vitro* was more rapid in *B*-*raf* mutant cell line COLO205, reflecting the increased sensitivity of *B*-*raf* mutated tumors to MEK inhibition. Data obtained by cellular fractionation and Western blotting suggest that the change of [^18^ F]FDG uptake associated with MEK inhibition might be due to translocation of GLUT1 from membrane to cytosol. A future study, using preclinical dynamic [^18^ F]FDG-PET imaging and kinetic parameters analysis in response to RO5126766 treatment and its correlation with our in vitro findings would be very interesting.

## Competing interests

The authors declare that they have no competing interests.

## Authors’ contributions

TT performed the *in vitro* FDG uptake studies, human tumor xenografts establishment, immunohistochemistry and drafted the manuscript; LiL assisted with *in vitro* and *ex*-*vivo studies*; TT, LiL, SSE carried out the *in vivo* FDG-PET imaging, data acquisition and analysis; NI, NH provided RO5126766 for the study and SOP of xenografts experiments; NI, NH, SSE, LuL, VM, JT and PP participated in the study design and coordination. All authors read and approved the final manuscript.

## Supplementary Material

Additional figure 1: Figure S1 The comparison of basal glucose utilization by the three human colon carcinoma cell lines. Radioactivity uptake is normalized to the total protein content. Click here for file

## References

[B1] Friday BB Adjei AA Advances in targeting the Ras/Raf/MEK/Erk mitogen-activated protein kinase cascade with MEK inhibitors for cancer therapy Clin Cancer Res 2008 3 342 346 10.1158/1078-0432.CCR-07-4790 18223206

[B2] Sullivan RJ Atkins MB Molecular targeted therapy for patients with melanoma: the promise of MAPK pathway inhibition and beyond Expert Opin Investig Drugs 2010 3 1205 1216 10.1517/13543784.2010.504709 20687784

[B3] Pratilas CA Solit DB Targeting the mitogen-activated protein kinase pathway: physiological feedback and drug response Clin Cancer Res 2010 3 3329 3334 10.1158/1078-0432.CCR-09-3064 20472680 PMC2912210

[B4] LoRusso PM Krishnamurthi SS Rinehart JJ Nabell LM Malburg L Chapman PB DePrimo SE Bentivegna S Wilner KD Tan W Ricart AD Phase I pharmacokinetic and pharmacodynamic study of the oral MAPK/ERK kinase inhibitor PD-0325901 in patients with advanced cancers Clin Cancer Res 2010 3 1924 1937 10.1158/1078-0432.CCR-09-1883 20215549

[B5] O'Neil BH Goff LW Kauh JS Strosberg JR Bekaii-Saab TS Lee RM Kazi A Moore DT Learoyd M Lush RM Phase II study of the mitogen-activated protein kinase 1/2 inhibitor selumetinib in patients with advanced hepatocellular carcinoma J Clin Oncol 2011 3 2350 2356 10.1200/JCO.2010.33.9432 21519015 PMC3107750

[B6] Kim KB Kefford R Pavlick AC Infante JR Ribas A Sosman JA Fecher LA Millward M McArthur GA Hwu P Phase II study of the MEK1/MEK2 inhibitor Trametinib in patients with metastatic BRAF-mutant cutaneous melanoma previously treated with or without a BRAF inhibitor J Clin Oncol 2013 3 482 489 10.1200/JCO.2012.43.5966 23248257 PMC4878037

[B7] Turajlic S Ali Z Yousaf N Larkin J Phase I/II RAF kinase inhibitors in cancer therapy Expert Opin Investig Drugs 2013 3 739 749 10.1517/13543784.2013.797964 23642225

[B8] Solit DB Santos E Pratilas CA Lobo J Moroz M Cai S Blasberg R Sebolt-Leopold J Larson S Rosen N 3'-deoxy-3'-[18F]fluorothymidine positron emission tomography is a sensitive method for imaging the response of BRAF-dependent tumors to MEK inhibition Cancer research 2007 3 11463 11469 10.1158/0008-5472.CAN-07-2976 18056475 PMC3203690

[B9] Su H Bodenstein C Dumont RA Seimbille Y Dubinett S Phelps ME Herschman H Czernin J Weber W Monitoring tumor glucose utilization by positron emission tomography for the prediction of treatment response to epidermal growth factor receptor kinase inhibitors Clin Cancer Res 2006 3 5659 5667 10.1158/1078-0432.CCR-06-0368 17020967

[B10] Sohn HJ Yang YJ Ryu JS Oh SJ Im KC Moon DH Lee DH Suh C Lee JS Kim SW [18F]Fluorothymidine positron emission tomography before and 7 days after gefitinib treatment predicts response in patients with advanced adenocarcinoma of the lung Clin Cancer Res 2008 3 7423 7429 10.1158/1078-0432.CCR-08-0312 19010859

[B11] Schelling M Avril N Nahrig J Kuhn W Romer W Sattler D Werner M Dose J Janicke F Graeff H Schwaiger M Positron emission tomography using [(18)F]Fluorodeoxyglucose for monitoring primary chemotherapy in breast cancer J Clin Oncol 2000 3 1689 1695 10764429 10.1200/JCO.2000.18.8.1689

[B12] Stroobants S Goeminne J Seegers M Dimitrijevic S Dupont P Nuyts J Martens M van den Borne B Cole P Sciot R 18FDG-Positron emission tomography for the early prediction of response in advanced soft tissue sarcoma treated with imatinib mesylate (Glivec) Eur J Cancer 2003 3 2012 2020 10.1016/S0959-8049(03)00073-X 12957455

[B13] Weber WA Petersen V Schmidt B Tyndale-Hines L Link T Peschel C Schwaiger M Positron emission tomography in non-small-cell lung cancer: prediction of response to chemotherapy by quantitative assessment of glucose use J Clin Oncol 2003 3 2651 2657 10.1200/JCO.2003.12.004 12860940

[B14] McCubrey JA Steelman LS Chappell WH Abrams SL Franklin RA Montalto G Cervello M Libra M Candido S Malaponte G Ras/Raf/MEK/ERK and PI3K/PTEN/Akt/mTOR cascade inhibitors: how mutations can result in therapy resistance and how to overcome resistance Oncotarget 2012 3 1068 1111 23085539 10.18632/oncotarget.659 PMC3717945

[B15] Belden S Flaherty KT MEK and RAF inhibitors for BRAF-mutated cancers Expert reviews in molecular medicine 2012 3 17 10.1017/erm.2012.11 23058743

[B16] McArthur GA Puzanov I Amaravadi R Ribas A Chapman P Kim KB Sosman JA Lee RJ Nolop K Flaherty KT Marked, homogeneous, and early [18F]fluorodeoxyglucose-positron emission tomography responses to vemurafenib in BRAF-mutant advanced melanoma J Clin Oncol 2012 3 1628 1634 10.1200/JCO.2011.39.1938 22454415 PMC5950495

[B17] Leijen S Middleton MR Tresca P Kraeber-Bodere F Dieras V Scheulen ME Gupta A Lopez-Valverde V Xu ZX Rueger R Phase I Dose-Escalation Study of the Safety, Pharmacokinetics, and Pharmacodynamics of the MEK Inhibitor RO4987655 (CH4987655) in Patients with Advanced Solid Tumors Clin Cancer Res 2012 3 4794 4805 10.1158/1078-0432.CCR-12-0868 22767668

[B18] Martinez-Garcia M Banerji U Albanell J Bahleda R Dolly S Kraeber-Bodere F Rojo F Routier E Guarin E Xu ZX First-in-Human, Phase I Dose-Escalation Study of the Safety, Pharmacokinetics, and Pharmacodynamics of RO5126766, a First-in-Class Dual MEK/RAF Inhibitor in Patients with Solid Tumors Clin Cancer Res 2012 3 4806 4819 10.1158/1078-0432.CCR-12-0742 22761467

[B19] Lyrdal D Boijsen M Suurkula M Lundstam S Stierner U Evaluation of sorafenib treatment in metastatic renal cell carcinoma with 2-fluoro-2-deoxyglucose positron emission tomography and computed tomography Nuclear medicine communications 2009 3 519 524 10.1097/MNM.0b013e32832cc220 19522059

[B20] Leyton J Smith G Lees M Perumal M Nguyen QD Aigbirhio FI Golovko O He Q Workman P Aboagye EO Noninvasive imaging of cell proliferation following mitogenic extracellular kinase inhibition by PD0325901 Mol Cancer Ther 2008 3 3112 3121 10.1158/1535-7163.MCT-08-0264 18790789

[B21] Engelman JA Chen L Tan X Crosby K Guimaraes AR Upadhyay R Maira M McNamara K Perera SA Song Y Effective use of PI3K and MEK inhibitors to treat mutant Kras G12D and PIK3CA H1047R murine lung cancers Nat Med 2008 3 1351 1356 10.1038/nm.1890 19029981 PMC2683415

[B22] Vergez S Delord JP Thomas F Rochaix P Caselles O Filleron T Brillouet S Canal P Courbon F Allal BC Preclinical and clinical evidence that Deoxy-2-[18F]fluoro-D-glucose positron emission tomography with computed tomography is a reliable tool for the detection of early molecular responses to erlotinib in head and neck cancer Clin Cancer Res 2010 3 4434 4445 10.1158/1078-0432.CCR-09-2795 20660574

[B23] Cullinane C Dorow DS Kansara M Conus N Binns D Hicks RJ Ashman LK McArthur GA Thomas DM An in vivo tumor model exploiting metabolic response as a biomarker for targeted drug development Cancer Res 2005 3 9633 9636 10.1158/0008-5472.CAN-05-2285 16266981

[B24] Ishii N Harada N Joseph EW Ohara K Miura T Sakamoto H Matsuda Y Tomii Y Tachibana-Kondo Y Iikura H Enhanced inhibition of ERK signaling by a novel allosteric MEK inhibitor, CH5126766, that suppresses feedback reactivation of RAF activity Cancer Res 3 13 4050 4060 10.1158/0008-5472.CAN-12-3937 PMC4115369 23667175

[B25] Kraeber-Bodere F Carlier T Naegelen VM Shochat E Lumbroso J Trampal C Nagarajah J Chua S Hugonnet F Stokkel M Differences in the biologic activity of 2 novel MEK inhibitors revealed by 18F-FDG PET: analysis of imaging data from 2 phase I trials J Nucl Med 2012 3 1836 1846 10.2967/jnumed.112.109421 23143089

[B26] Samen E Thorell JO Lu L Tegnebratt T Holmgren L Stone-Elander S Synthesis and preclinical evaluation of [(11)C]PAQ as a PET imaging tracer for VEGFR-2 Eur J Nucl Med Mol Imaging 2009 3 1283 1295 10.1007/s00259-009-1111-3 19288096

[B27] Fueger BJ Czernin J Hildebrandt I Tran C Halpern BS Stout D Phelps ME Weber WA Impact of animal handling on the results of 18F-FDG PET studies in mice J Nucl Med 2006 3 999 1006 16741310

[B28] Avril N GLUT1 expression in tissue and (18)F-FDG uptake J Nucl Med 2004 3 930 932 15181126

[B29] Songji Z Yuji K al e Biologic Correlates of Intratumoral Heterogeneity in 18F-FDG Distribution with Regional Expression of Glucose Transporters and Hexokinase-II in Experimental Tumor J Nucl Med 2005 3 675 682 15809491

[B30] Gatenby RA Gillies RJ Why do cancers have high aerobic glycolysis? Nat Rev Cancer 2004 3 891 899 10.1038/nrc1478 15516961

[B31] Chung JK al e Mechanisms related to (18F) Fluorodeoxyglucose uptake of human coln cancers transplanted in nude mice J Nucl Med 1999 3 339 346 10025844

[B32] Yun J Rago C Cheong I Pagliarini R Angenendt P Rajagopalan H Schmidt K Willson JK Markowitz S Zhou S Glucose deprivation contributes to the development of KRAS pathway mutations in tumor cells Science 2009 3 1555 1559 10.1126/science.1174229 19661383 PMC2820374

[B33] Brepoels L Stroobants S Verhoef G De Groot T Mortelmans L De Wolf-Peeters C (18)F-FDG and (18)F-FLT uptake early after cyclophosphamide and mTOR inhibition in an experimental lymphoma model J Nucl Med 2009 3 1102 1109 10.2967/jnumed.109.062208 19525456

[B34] Kinross KM Brown DV Kleinschmidt M Jackson S Christensen J Cullinane C Hicks RJ Johnstone RW McArthur GA In vivo activity of combined PI3K/mTOR and MEK-inhibition in a KrasG12D;Pten deletion mouse model of ovarian cancer Mol Cancer Ther 3 8 1440 1449 10.1158/1535-7163.MCT-11-0240 21632463

[B35] Soret M Bacharach SL Buvat I Partial-volume effect in PET tumor imaging J Nucl Med 2007 3 932 945 10.2967/jnumed.106.035774 17504879

[B36] Bergstrom M Awad R Estrada S Malman J Lu L Lendvai G Bergstrom-Pettermann E Langstrom B Autoradiography with positron emitting isotopes in positron emission tomography tracer discovery Mol Imaging Biol 2003 3 390 396 10.1016/j.mibio.2003.09.004 14667493

[B37] Fritz V Fajas L Metabolism and proliferation share common regulatory pathways in cancer cells Oncogene 2010 3 4369 4377 10.1038/onc.2010.182 20514019 PMC3004916

[B38] Levine AJ Puzio-Kuter AM The control of the metabolic switch in cancers by oncogenes and tumor suppressor genes Science 2010 3 1340 1344 10.1126/science.1193494 21127244

[B39] DeBerardinis RJ Lum JJ Hatzivassiliou G Thompson CB The biology of cancer: metabolic reprogramming fuels cell growth and proliferation CELL METAB 2008 3 11 20 10.1016/j.cmet.2007.10.002 18177721

[B40] Bading JR Shields AF Imaging of cell proliferation: status and prospects J Nucl Med 2008 3 Suppl 2 64S 80S 18523066 10.2967/jnumed.107.046391

