# Supplementary figures and images for: [18 F]FDG-PET imaging is an early non-invasive pharmacodynamic biomarker for a first-in-class dual MEK/Raf inhibitor, RO5126766 (CH5126766), in preclinical xenograft models
Source: EJNMMI Res. 2013 Sep 16;3:67. doi: 10.1186/2191-219X-3-67 (PMC3848680; doi:10.1186/2191-219X-3-67)

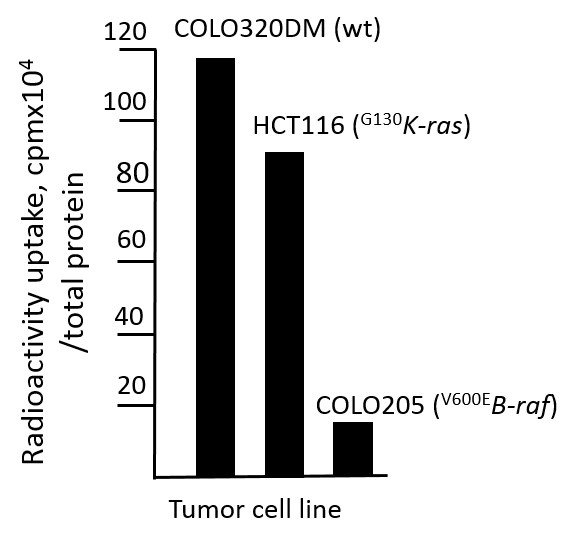

Supplement: Additional figure 1: Figure S1 — The comparison of basal glucose utilization by the three human colon carcinoma cell lines. Radioactivity uptake is normalized to the total protein content. [file 2191-219X-3-67-S1.jpeg]
